# Sentencing Policy, Social Values and Discretionary Justice

**DOI:** 10.1093/ojls/gqac011

**Published:** 2022-06-07

**Authors:** Ralph Henham

**Keywords:** sentencing policy, social justice, judicial discretion, restorative justice

## Abstract

Despite the recent consolidation of sentencing law and procedure, the fundamental values which underpin the policy and practice of sentencing in England and Wales have remained largely unchanged since the deserts-based model introduced by the Criminal Justice Act of 1991. It is argued that this paradigm is no longer appropriate and presents a significant impediment to reducing imprisonment and mainstreaming restorative forms of intervention within the criminal process. An alternative value-based approach is proposed to counter this trend, one that provides greater structural flexibility and empowers sentencers to engage more effectively with the social impact of penal intervention.

## 1. Introduction

The values which underpin sentencing policy are of enormous symbolic and practical significance since they provide both the moral authority for state punishment and inform the ethics and practice of sentencing. Such values do not exist in isolation; they are fundamental in affirming and delineating the moral relationship between citizen and state. In effect, they represent the ‘terms’ upon which individual liberty and state power are configured with regard to punishment.

Values are constantly shifting and vary over time. Thus, they may be used to justify increased state control through harsh and punitive measures designed to subjugate and repress those who threaten the status quo. In such circumstances, the extent to which the state is willing to cede discretionary power to the judiciary in sentencing matters may be significantly reduced. Moreover, social factors which influence criminality and impact the social effectiveness of penal measures may be marginalised where the exercise of sentencing discretion is reduced or narrowly prescribed. The values which underpin sentencing policy are therefore key in establishing the degree to which the judiciary is able to exert any practical influence over the penal regime the state pursues. Since such values set the moral parameters for permissible judicial intervention, an impartial and independent judiciary is a basic prerequisite to protect citizens against discriminatory and harsh punishment.^1^

This article argues that fundamental change in the values which justify punishment and inform the sentencing policy and practice of England and Wales is long overdue.^2^ In broad terms, this involves a move away from the dominant retributive focus towards values which promote a different conception of the role of punishment within civil society: one that envisages punishment as having the unifying social purpose of promoting social cohesion. Such a paradigm shift focuses on two essentially interrelated issues:

1. A fundamental reappraisal of punishment’s moral justification as currently represented by the state; and, in consequence,
2. Profound ethical and normative changes in sentencing policy and practice, to facilitate the integration of restorative solutions into mainstream criminal trial practice.

Against this background, the article examines the concept of judicial discretionary power and explores its socio-legal significance for sentencing policy and practice. The degree of judicial discretionary power in sentencing is conceived as having immense symbolic and practical significance in both articulating and operationalising penal values and as playing a potentially key role in strengthening social cohesion. A particular focus in this context is Durkheim’s notion of moral individualism^3^ and his writings about the relationship between penal values and social solidarity.^4^ This analysis prompts a deeper exploration of the relationship between contemporary sentencing policy and social cohesion, focusing particularly on the impact of value pluralism.

What follows aims to chart a different moral course for punishment and the future direction of sentencing policy and practice. The argument is developed through an analysis of contemporary debates about the nature of discretionary justice and its limited capacity to fulfil citizens’ expectations for ‘justice’. The discussion then broadens to address the impact of increased value pluralism and the importance for social cohesion of establishing a coherent relationship between the values that inform penal ideology and social morality.

The article then discusses the conceptual limitations of the present sentencing paradigm in responding to these challenges, before moving on to explain the need for a fresh conceptualisation of discretionary justice, expanding the idea of justice as having both individual *and* social dimensions, and exploring what that might signify for the moral credibility of sentencing. The discussion considers the pivotal role of judicial discretionary power in mediating the changing moral relationship between citizen and state as regards punishment, before exploring how these arguments relate to the issue of devolved or increased local accountability for criminal justice. It then suggests how a moral understanding of the ‘public interest’ might be advanced to inform sentencing.

Finally, the article explains how the use of judicial discretionary power in sentencing could be developed as a communitarian practice which enhances social rehabilitation and reintegration through restorative intervention. It concludes that a penality^5^ which reflects shared values would help to increase social cohesion and promote ‘social justice’.^6^

## 2. Discretionary Justice and Social Values

The relationship between judicial discretionary power in sentencing and social values is infinitely complex.^7^ The origins and meaning of ‘discretion’ in sentencing, the concept and exercise of judicial discretionary power and citizens’ perceptions of what constitutes discretionary ‘justice’ are interrelated issues. ‘Discretion’ implies a degree of individual freedom in judicial decision making with a capacity to interpret or deploy sentencing law and procedure in reaching innovative or constructive solutions.^8^ However, the nature of discretionary decision making in sentencing, how decisions are justified and the contextual influences that inform them remain unclear.^9^

Such debates are important in drawing our attention to the idea that the relationship between state power and sentence decision making is pivotal in shaping our understanding of how citizens, both individually and collectively, perceive punishment. In theory, the sentencing of offenders may be conceived as a state-sponsored activity where power is exercised institutionally through the punishment of convicted offenders on behalf of *all* citizens. The latter imbues state punishment with a particular kind of moral authority. Thus, one may argue that citizen and state should share similar penal values if trust and the legitimacy of institutionalised punishment are to be established and sustained.^10^ A key component in establishing relationships of trust and legitimacy based on a common or shared morality is the idea that citizen and state share an attachment to similar values for the common good.

A belief in ‘social justice’ is fundamental to the notion of the common good.^11^ For present purposes, ‘social justice’ refers to the extent to which citizens collectively perceive the punishment of criminalised behaviour by the state as even-handed and non-discriminatory. Hence, citizens’ perceptions regarding the morality of the state’s approach to issues such as criminalisation or the impact of race, poverty and social deprivation on crime and punishment are all relevant. Where such perceptions are held collectively by citizens, notions of ‘social justice’ may be said to reflect a shared morality with shared values. However, it is important to note that shared values may also signify divergent or overlapping concerns and interests.

As a moral ideal, the use of punishment in the pursuit of ‘social justice’ could be described as virtuous because it is more likely to foster social cohesion than conflict. This implies that ‘social justice’ has intrinsic value as a morality that tends to promote the common good.^12^ However, the values a state *might* adopt to foster such a shared morality always remain a matter for conjecture.^13^ Accordingly, the extent to which norms and practices based on shared values *actually* contribute to social cohesion is relative to time and place.

Durkheim argues that the morality attached to punishment is a function of social conditions.^14^ Cotterrell describes Durkheim’s sociology of justice as based on the idea of justice as necessary for achieving and maintaining social solidarity; it is concerned with identifying shared understandings (a collective consciousness) that citizens must internalise for society to exist as a moral unity.^15^ Durkheim identifies the notion of ‘moral individualism’^16^ as essential to the collective consciousness of modern industrialised societies because it elevates the dignity and autonomy of each individual as a moral priority. Thus, Durkheim sees moral evolution and hence the social significance attached to moral values such as moral individualism as the result of the interplay between social and personal forces. Consequently, state punishment may be conceived as reflecting shared sentiments that the wrongdoer has offended in the particular context of the offence and as having a wider function of reinforcing the social value placed upon those shared sentiments.^17^

Notwithstanding, as Cotterrell explains,^18^ Durkheim’s functionalism prevents him from taking proper account of the subjective experiences and meanings of social solidarity and, crucially, of the fact that *in reality* group relationships may not be organised for the common good. Therefore, Durkheim does not engage with some of the more significant empirical questions concerned with developing a shared morality to promote the common good. Hence, Durkheim’s account of social solidarity falls short of providing a fully convincing explanation of the origins and content of social morality, since it takes no account of how values are shaped by social experiences. Whereas, in an abstract sense, moral individualism may retain importance as a unifying thread, it has no tangible existence in the real world. Rather, the extent to which citizens *actually* share values of respect for human dignity is seen predominantly as a function of social conditions. However, a crucial gap in our understanding concerns the way in which experiences of criminal justice, and sentencing in particular, shape individual and shared perceptions of morality within particular social contexts.^19^

In sum, Durkheim’s functionalism does not help us to bridge the gap between individual morality and the manifestion of a collective conscience. Although a value system based on moral individualism may be conceptualised as key to promoting a shared morality that sustains ‘social justice’, this would require empirical verification. Moreover, where value pluralism exists, there may be no common bonds or mutual dependency, and, therefore, no identifiable social morality. Social morality in the late modern era is likely to be more fragmented than shared, thereby increasing the moral distance between notions of state-sponsored ‘justice’ and citizens’ perceptions. This lacuna may grow where sentence decision making is subject to prescriptive guidelines which restrict the possibilities for individualised sentencing.

## 3. Value Pluralism and Social Cohesion

It has been argued that penal ideology, policy and practice should be invested with a particular kind of moral authority whereby citizens share a belief in the moral virtue of state punishment as a response to crime.^20^ However, in reality, this moral authority may be lacking where state penality operates within a climate of moral obfuscation with competing, contradictory or overlapping values and interests. Such a penal environment is likely to erode trust between citizen and state, pre-empting a gradual withdrawal of legitimacy from state punishment.

The late modern era has witnessed increasing secularisation^21^ and a general polarisation of moral values, aggravated by the absence of any sustained debate about the kinds of values and norms which are (or should be) shared generally by those who have a moral stake in promoting social harmony.^22^ In such circumstances, institutionalised processes such as sentencing are more likely to encourage social unrest if they consistently re-enforce notions of responsibility that appear remote from the social reality of many citizens’ daily lives; for example, by marginalising the impact of poverty and social deprivation on criminality.

This phenomenon may have wider repercussions. Lack of moral empathy with the values and purposes of state punishment may reflect a shared perception that sentencing is implicated in reproducing the social injustices of unfair criminalisation, particularly where criminality is closely associated with abnormally high levels of poverty and social deprivation. Punishment may also be perceived as reflecting wider social injustices which criminalise and penalise the opinions and practices of secular and religious groups whose values do not reflect those adopted by the state.^23^ Penal values are unlikely to reflect what the state presumes to be the values of the majority of citizens, particularly where value pluralism persists. Accordingly, the state’s moral authority in penal matters typically rests upon somewhat fragile foundations, a situation which may be readily compounded since the state has the power to ‘interpret’ social morality. Thus, the moral virtue of state penality and that of its penal institutions cannot be assumed; what this signifies in social terms must be verified empirically.

The relationship between value pluralism and the perceived legitimacy of punishment has considerable significance. To argue that value pluralism tends to distort *accepted* notions of responsibility for crime and punishment is to presume that the existing law and practice of sentencing reflects some kind of moral consensus about the meaning of ‘responsibility’ within specific social contexts. This is self-evidently not the case. State penality has always mirrored the uneasy and volatile relationship that exists between the powerful and the subjugated.^24^ Hence, the way punishment is justified morally by the state always reflects a socially sensitive judgment about who should bear the moral responsibility for crime, the criminalisation of certain behaviours and the severity of the penal response.

Durkheim’s notion of ‘justice’ depends largely on the extent to which *all* citizens are able to maximise their social and economic interests.^25^ However, such interests can only be pursued within a society that allows social and economic relationships to flourish. According to Durkheim, this kind of society requires the social solidarity of a shared value system supported by the state, one which recognises and tolerates differences in culture and practice. Thus, the need for shared values becomes more acute within morally pluralistic societies as social and economic relations become more complex and fragmented. Cotterrell argues that the morality which dictates legal and moral values, and so determines the nature and scope of individual rights and responsibilities, depends upon the stability and predictability of society’s solidarity and unity.^26^ Durkheim’s notion of moral individualism is proposed as the value system that is most likely to promote the autonomy and dignity of the individual in societies where value pluralism persists.^27^

Attacks on the dignity and autonomy of the individual, such as hate crime, may be regarded as significant attacks on the value system of moral individualism. Although Durkheim places considerable emphasis on moral individualism at the expense of other forms of attachment demanded by culture, religion or class, social solidarity may suggest different reasons for adherence than moral individualism.^28^ Whilst Durkheim signifies the social value of moral individualism as promoting social solidarity, it is important to remember that the normative effect of moral individualism is always relative. In essence, the social value of any link between moral individualism and social solidarity can only be determined by explaining its context. Although one might accept that moral individualism has a crucial role to play in promoting social solidarity, and is desirable for this reason, this should not detract from the fact that individual autonomy and dignity are desirable values per se and should be universally recognised as such.^29^

Finally, it is important to recognise the role of psychological variables such as the emotions^30^ in shaping individual and shared perceptions about the value of punishment and sentencing. This level of understanding involves deconstructing complex psychological processes, particularly the way moral empathy with particular forms of penal resolution might develop. A profound appreciation of this reality is fundamental in explaining why certain penal values predominate and in deciding how the penal relationship between citizen and state might be sustained in the future.^31^

## 4. Social Values, Social Justice and Politics

State ideology defines the conceptual link between social morality and penal justifications. As suggested, the way the state justifies the penal response to criminality rests upon constantly shifting moral foundations. Moreover, the state’s moral reasoning is frequently unclear. Historically, the state’s ability to exert power and maintain social control has signified the moral value of punishment, with the imposed morality of the dominant elite likely to conflict with that of citizens who feel oppressed and socially excluded. Hence, the abstract values that inform penal policy and practice may lack either temporal or contextual validity.^32^

It has been argued that the perceived value of the state’s actions, and hence its moral authority to punish, depends upon its ability to engage with citizens in a moral sense. Koch suggests that an anthropological approach is necessary to fully comprehend the basis of the state’s moral authority, arguing that a fully democratic politics of criminal justice requires far greater engagement with the most subordinated citizens.^33^ Moreover, Koch maintains that certain socially deprived communal groups develop their own hidden moralities and justifications for action, which, paradoxically, appear to intensify the more the state attempts to exert localised control over citizens’ lives. Koch therefore advocates a more citizen-centred understanding of localised morality within subordinated communal groups, one aimed at delivering penal policies that are better equipped to link state morality with the moral responses of citizens. Naturally, this becomes more difficult as the moralities of state and citizen diverge.

Following the demise of the paternalistic state,^34^ moral ambiguity has increasingly characterised penal justifications in late modernity.^35^ In addition, value pluralism has obfuscated the social value of punishment and weakened the legitimacy attached to the institutions of criminal justice.^36^ As Koch implies, the more state punishment is perceived as a tool of political oppression, the greater the propensity for social conflict and breakdown in social cohesion. Paradoxically, in imposing a dominant penal morality to achieve conformity, opposing moralities may either be strengthened or marginalised.^37^ As a consequence, social cohesion may be weakened. To counter this, penal morality should be contextualised and socially embedded to reflect the complexity of modern social relations, particularly how those relationships represent overlapping and interdependent networks or community interests.^38^ In essence, this novel approach requires a localised and contextualised morality.

Various options present themselves. ‘Democratising’ punishment by importing public opinion into sentencing does not deal adequately with the reality that social injustice is structurally embedded. Ironically, such an approach may unintentionally reinforce social inequality. Deserts-based sentencing systems, on the other hand, tend to amplify social inequality by marginalising evidence relating to the impact of poverty and social deprivation on criminality.^39^ Whereas sentencing policy alone cannot rectify structural inequality,^40^ existing penal values are instrumental in reinforcing the status quo of social relations. For example, criminalisation and punishment is increasingly perceived by the BAME community as discriminatory. Although the determination of offence ‘seriousness’ may be aggravated by racial motive for sentencing purposes, accountability through the sentencing process is constrained by what are fundamentally retributive values and norms. Hence, the narrow focus on individual culpability and harm precludes any greater engagement with the social impact of sentencing outcomes.^41^ A penal ideology based on values which promote social justice in sentencing would help to reverse this trend. However, significant moral consensus between citizen and state as to the justifications and purposes for punishment would be required to validate such a change and facilitate the ethical and normative structures needed to operationalise this aspiration.

However, as noted, achieving greater social justice in sentencing will crucially depend upon the development of judicial discretionary power. With changed priorities and choices available, sentencers would be empowered to take greater account of punishment’s social impact.^42^ Moreover, increased emphasis on the social dimensions of accountability could increase communitarian forms of penal intervention aimed at social rehabilitation. Hence, redefining the boundaries of the penal relationship between citizen and state by reducing the moral distance between them should bolster the state’s moral authority to punish and help to promote social justice in sentencing.

## 5. Conceptualising Discretionary Justice

As suggested, the term ‘discretion’ implies a degree of freedom and capacity in decision making,^43^ whereas the notion of ‘justice’ ascribes value to the decisions themselves. ‘Justice’ has interrelated individual and social dimensions whose social meaning should be contextualised relative to time and place. Accordingly, notions of ‘discretionary justice’ should reflect the extent to which sentencers are able to take account of factors which impact the social value of sentencing decisions; for example, the relative impact of social deprivation on criminality. This approach requires a meaningful engagement by decision makers with the moral and social contexts of crime and punishment.

The nature and exercise of discretion in sentencing has received recent scholarly attention.^44^ Anleu, Brewer and Mack conclude that ‘sentencing necessarily involves striking an intricate balance fusing emotion and legal-rational requirements within the confines of the complex world of sentencing’.^45^ However, it may be argued that this approach tends to oversimplify our understanding of sentencing. There is much discussion about the methodological limitations of legal positivism and those of empirically focused quantitative approaches, questioning their ‘capacity to provide rich explanations for judicial sentencing behaviour, or to examine judicial subjectivity’.^46^

Such assertions may be questioned. To begin with, there is a tendency for some commentators to simply describe sentencing ‘law’ as ‘rules’ without further explanation. There is a sense, of course, in which this is true—law is normative. However, it is also true that law has intrinsic qualities that distinguish it from other ‘rules’, such as rules prescribing social behaviour. Thus, law is analysed as a distinct category of ‘rules’—law demands precision and certainty, as does its understanding. The only fixed factor in sentencing is the applicable law.^47^ The sentencing principles established by the Court of Appeal Criminal Division and the sentencing guidelines produced by the Sentencing Council for England and Wales differ both in status and intended consequences. Thus, it is necessary to appreciate the interrelationship of law and decision maker, be it judge or magistrate, not simply as socio-legal interaction, but, equally important, as a context where the ‘meaning’ of law itself is established.^48^ Hence, neither doctrinal legal analysis nor legal positivism should be marginalised or subsumed within a generalised sociological categorisation. Although research should focus on the wider social and moral contexts of sentencing, a profound analysis of law’s meaning within the context of other analytical perspectives is necessary to fully comprehend its role in decision making.

Judges are both constrained and unconstrained in terms of how they interact with legal and social contexts. Hogarth attempts to account for the impact of this dynamic on the exercise of judicial discretionary power.^49^ However, judicial emotional engagement is not simply restricted to managing processes, interacting with courtroom participants or reacting to the social world beyond the courtroom—it is integral to legal interaction and the exercise of discretionary power in sentencing. Thus, one might caution against distinguishing the judicial approach to law from interaction with other social norms and processes. Explaining how and why the emotions and legal-rationality are interrelated is essential to provide a fully social account of sentencing.

Notwithstanding, the comprehension and interpretation of sentencing law, principle and guidance does not depend solely upon the emotional sensibilities of the person seeking the information. As Cotterrell points out, the ‘communicable knowledge of legal doctrine’ does exist. So legal reasoning does not exist in isolation; it must have some kind of creative purpose.^50^ Whilst the nature of legal knowledge and the skills required to access it are beyond the scope of this article, its distinctive existence should be acknowledged when analysing sentencing from a socio-legal or sociological perspective.

How judges interact with, rationalise, interpret and apply the law in any particular case has interrelated moral and social dimensions.^51^ As Duff^52^ and Garland^53^ emphasise, law is a communicative enterprise, which, in the context of penal law, means that it is concerned with conveying certain information to citizens about the penal consequences of criminalised behaviour. Sentencing law normally sets out specific penal measures and commonly refers to the purposes of punishment.^54^ Both have moral significance. Fundamentally, sentencing law reflects a moral judgment about the behaviour in question and what the state regards as the appropriate parameters for punishing that behaviour at a particular moment in time.^55^ How that morality is perceived is intrinsic to the communicative process that takes place between citizen and state each time a sentencing decision is made. So the morality sentencing law represents is an idealised morality; it endorses a particular moral position on punishment against which criminal behaviour must be judged.

However, the morality which underpins sentencing law provokes differing emotional reactions when operationalised through the medium of a sentencing decision. The relationship between judge and offender expressed in sentencing has a distinct and individualised moral quality. Hence, particular emotional sensibilities are engaged when, following conviction, penal purposes are operationalised through sentencing. Furthermore, the interactive context in which judicial discretionary power is exercised within the decision-making process is dictated by sentencing policy. This context is significant in influencing the extent to which state values are likely to be realised through the sentence decision-making process and beyond. Viewed thus, moral values reflect how the state wishes crime to be resolved through the criminal process. What constitutes ‘fact’ and, ultimately, ‘truth’ is filtered through laws, rules and procedures designed to operationalise underlying values and purposes, all of which are contextualised through practice.

Tata repeatedly refers to ‘rules’ and the rule–fact dichotomy, particularly the indeterminacy of rules and their reliance on facts which are produced by the process.^56^ Whilst Tata appears to interpret the meaning of ‘rules’ sociologically as guides to conduct, this does not distinguish adequately between the ‘rules’ of sentencing: namely, sentencing law contained in legislation; sentencing principles delivered by the higher judiciary sitting in the Court of Appeal Criminal Division; sentencing guidelines promulgated by the Sentencing Council for England and Wales; rules of procedure relating to trial and sentence, such as the Criminal Procedure Rules and Practice Directions; or the relevant law of evidence and its associated procedural rules. Such ‘rules’ are more or less prescriptive and differ markedly in the extent to which they are ‘fact’-dependent.^57^ Hence, ‘rules’ are not always ambiguous and indeterminate so that they can be described as ‘produced’ by the process, as Tata suggests.

Although Tata acknowledges that process signifies something other than procedure,^58^ he focuses on the idea that process is largely concerned with building an agenda for the purpose of sentencing. In so doing, he highlights the impact of pre-trial process, especially the guilty plea, the resulting avoidance of the trial stage and the processual emphasis of sentencing. Moreover, Tata stresses the ‘socially-purposive account giving nature of sentencing’ and the idea that sentences are deliberately crafted to satisfy particular audiences or constituences.^59^ He states that ‘[by] “socially effective” I mean accounts whose messages are understood as intended by the different intended audiences’. It may be questioned whether Crown Court judges deliberate the need to satisfy different audiences in this way. Thinking about ‘accounting for sentencing as a kind of performance of balancing competing interests and values’ may be part of what a sentencer does,^60^ but this ignores the wider question of why and how particular values should take priority over others and the impact of this on sentencing practice and beyond. Ultimately, moral and political factors inform the way sentencing is ‘communicated and understood’. These are not matters for sentencers. The state decides how the penal relationship between citizen and state ought to be configured and, in particular, the moral distance between them.

The process of sentencing consists of a series of socially interactive stages circumscribed by procedural rules. Hence, the extent to which judges are able to balance or reconcile competing interests or values in particular ways is both a function of the process *and* its underlying moral purpose(s), rather than something that depends solely upon the degree of unfettered judicial discretionary power. Within such constraints, sentencers must seek to justify the moral virtue of punishment in the individual case.^61^ Whilst procedural rights and due process are an important aspect of this, understandings of what rights and access to justice mean in reality need to be contextualised. Ethical practice does not function in social isolation. Procedural justice and adherence to due process norms are significant factors in developing the kind of communitarian trust needed for sentencing to become a credible platform for restorative intervention. However, the state has a moral obligation to justify such interventions for the common good on the basis of identifiable and shared social values.^62^

## 6. Discretionary Justice and the Changing Relationship between Citizen and State

Judicial discretionary power is instrumental in operationalising sentencing policy and, in so doing, reaffirms the values and justifications for punishment adopted by the state. Du Bois-Pedain identifies morality as a central concern in conceptualising the relationship between citizen and state.^63^ Moreover, she makes the point that the classical liberal ‘means to an end’ objection to reformatory punishment is misconceived, since the concept of freedom is relative. What is regarded as a fair and proportionate response to offending behaviour varies according to time and place. Hence, neither the justifications for criminalisation nor punishment itself lend themselves to precise definition. As du Bois-Pedain puts it, the ‘challenge is based on a certain conception of what respecting freedom means’.^64^ This challenge is one that seeks to identify common ground between the state’s criminal justice practices and citizens’ perceptions and expectations for ‘justice’. It has been argued that such perceptions should reflect values which are shared for reasons of the common good and that the state has a moral responsibility to identify and operationalise such values through policy and practice.^65^

Du Bois-Pedain believes that these understandings need to be shared by the population at large because they set the conditions that justify state punishment for all citizens, both now and for the future. Social cohesion in a Durkheimian sense may be threatened if some citizens feel that the penal state is not respecting their right to equal freedom. Accordingly, du Bois-Pedain considers that reformatory sentencing through individualisation demands constitutional authority, so that an offender’s punishment is based upon a constitutionally ‘legitimate’ proportionality assessment. Such an assessment would promote ‘core’ constitutional values of humanity and welfare. Hence, by linking proportionality to respect for the offender’s humanity, the state’s moral obligation to ensure that citizens are not disadvantaged through sentencing is consistent with the notion of social justice. This approach also resonates with Williams’s notion that sentencers should take responsibility for the consequences of punishing individual offenders.^66^

However, whilst the state has a moral obligation to promote social justice in order to fulfil this responsibility, this ideal does not represent social reality. Rather, the moral authority which the liberal constitutional state should draw from its citizens, and hence the values it uses to justify sentencing policy and practice, must be demonstrated empirically. Thus, the social value of punishment should find expression in determining sentencing policy and practice.

In supporting substantial sentencing discretion, du Bois-Pedain describes the sentencing experience as essentially a moral interaction wherein the sentencer seeks to establish ‘reasonable terms’ for the instant offence.^67^ Sentencing represents the basis upon which the polity’s ongoing relationship with the offender is to be progressed. It effectively personalises the state’s moral responsibility and recognises its fulfilment at the point of decision making, acknowledging the humanity and welfare of the offender in ‘a search for constructive punishments’. Hence, it is both ‘creative’ and ‘future-orienated’, but, above all, a ‘value-laden’ endeavour. In this context, du Bois-Pedain makes the crucial point that, notwithstanding structural constraints,^68^ the fact that the sentencer acknowledges moral responsibility for the outcome justifies the need for substantial sentencing discretion. Such moral responsibility is exercised on behalf of the polity and is to do with establishing the ‘reasonable terms’ which define the appropriate future relationship between the polity and the offender.

In sum, du Bois-Pedain argues that the framework for ‘constructive’ punishment should be set by law ‘in exercise of an authority conferred by the polity in the public interest’. This article argues for a moral understanding of the ‘public interest’, one that is evidenced by shared values for the common good. Such an understanding should validate the sentencing framework and guide sentencers in deciding what ‘reasonable terms’ might suggest themselves in the instant case.

## 7. Local Justice, Devolved Accountability and the ‘Public Interest’

Du Bois-Pedain describes the ‘public interest’ as linked conceptually to that of a collective political entity, and characterised by values generally associated with ‘civil society’—in other words, the archetypal liberal constitutional state.^69^ However, the social reality of the polity as a collective entity is difficult to visualise in terms of value pluralism, regional differences, cultural diversity and overlapping networks of community. Since the ‘public interest’ is a fluid and multidimensional concept, so are the values it seeks to represent. Linking the authority of the polity to the values of liberal democracy does not ensure social justice, although this may provide the constitutional authority and impetus for pursuing socially egalitarian policies.^70^

Hence, a moral understanding of the ‘public interest’ is proposed to fully comprehend the nature of what is, and should be, the ‘appropriate’ (contextualised) relationship between citizen and state as regards punishment, or what du Bois-Pedain refers to as ‘reasonable terms’ for state intervention. This section focuses on areas where this moral understanding of the ‘public interest’ currently appears to be either deficient or absent.

### A. Local Justice and Devolved Accountability

Increasingly, the social reality of devolved justice in England and Wales has been shaped by the politics of austerity, as exemplified by unprecedented court closures and funding cuts, and, more recently, a concerted drive towards the greater use of IT and so-called ‘virtual justice’. This tendency accelerated as a consequence of the social restrictions resulting from the ongoing coronavirus pandemic.

Nowhere have these effects been felt more acutely than in the magistrates’ courts, where the negative consequences of austerity have been compounded by similar policies affecting the efficiency of the Probation Service, the CPS, the police and the Prisons Service.^71^ Regrettably, as Riddle argues,^72^ the ideal paradigm of magistrates’ justice as locally focused and sensitive to community needs, values and interests has virtually disappeared. Whilst case management and the efficiency of trials has improved, procedural changes, reduced personnel, limited training and prescriptive sentencing guidelines have all conspired to diminish the value of summary justice. Notwithstanding, the argument against further circumscribing the discretionary powers of magistrates is more difficult to resist whilst recruitment and diversity issues remain unresolved. However, provided they are addressed, the case for local justice dispensed by magistrates who are demographically representative of the communities they serve remains compelling.

The morality through which sentencing policy is justified postulates notions of responsibility and accountability in relation to crime and punishment which may, or may not, reflect social reality.^73^ Most significant is the disjuncture between the values that purportedly justify state punishment and the increasingly diverse values of different ‘communities of interest’.^74^ The concept of ‘penal accountability’ therefore raises significant questions about the values that underpin the penal relationship between citizen and state, and, in particular, what this morality signifies for social cohesion at the local level. For instance, it seems reasonable to argue that the notion of ‘local justice’ should signify local accountability. If so, this raises the question of how state punishment can be justified in terms that are morally meaningful and accepted by local communities. This article has argued that state punishment should be informed by values that are shared for reasons of the common good. It follows that, in conceptualising and explaining the meaning of ‘local justice’, one should consider the degree to which values purporting to justify state punishment are shared at the local level. Such contextualised understandings should then reflect back into sentencing policy and practice, so that local imperatives, values and interests are always considered at sentencing, in terms of both substance and enforcement.

The rapid rise of IT and ‘virtual justice’ raises similar questions about the social value of punishment in terms of accountability. Tata describes judicial accountability as ‘socially-purposive account-giving’ rather than ‘simple line’ accountability,^75^ so drawing attention to the need for sentencers to satisfy conflicting or competing audiences. For this reason, it is important to recognise the limitations of algorithmic approaches in replicating the human process of judging^76^ and the significance of understanding and explaining punishment’s social purpose. It will become increasingly difficult to sustain the legitimacy of state punishment unless sentencing engages with contextualised understandings of social morality and takes account of the implications for sentence decision making and beyond. This is, in essence, a human endeavour.^77^

The coronavirus pandemic fast-tracked digital communication, providing virtual ‘coherence’ to criminal justice as the physical infrastructure began to disintegrate.^78^ Continuing political and economic instability is likely to enhance this trend, raising further questions about its potential impact on civil liberties, human rights and the operation of the courts.^79^ Thus, additional factors may be weighed together with the usual determinants of offence seriousness and victim harm in reaching a ‘proportionate’ response.^80^ Arguably, such additional pressures for system efficiency will further damage the credibility of an already overworked, underfunded and emaciated system of summary justice. Instead of helping to shape collective resolutions to crime for the common good, they do little to mediate the perceived injustices of the current regime.

More broadly, the pandemic challenged previously accepted justifications and structures of social relations and questioned existing ethics and practices. For sentencing, this suggests the need for a new, more egalitarian paradigm, combining virtual and physical processes, whereby locally valued solutions to crime and punishment are developed. A fundamental shift in underlying values, reconceptualising penal accountability as locally focused for the common good, would provide an opportunity to reconsider the justifications, practices and outcomes of sentencing. Informed by such values, sentencing might be reconceived as part of a socially rehabilitative process that widens access to community care and support, and hastens the desistance and reintegration of offenders.

### B. Procedural Justice v Social Justice

The increased focus on ‘process’ and ‘procedural justice’ resulting from the coronavirus pandemic may eventually lead to a transformation in the meaning of ‘substantive’ justice. However, such a trend has long been evident as bureaucratic and system efficiencies have gradually reshaped the meaning and significance attached to processual norms.^81^ Yet, there are no prescriptions for identifying which normative aspects of sentencing are associated with perceptions of procedural justice, or the extent to which these might be shared.

De Girolamo argues that procedural justice should be taken as a significant indicator of substantive justice, rather than other measures of justice such as ‘popular justice’.^82^ Exploring the distinctions between the constructs of legal and justice consciousness, De Girolamo observes that ‘justice consciousness … seeks subjective understandings of justice in particular contexts: the focus here is on the experience or perception of justice during a process [mediation] that sits outside of the law’. However, as argued, the moral subjectivity of ‘justice’ as everyday experience is difficult to reconcile with the objectivity of state penal practice as reflecting a core set of values through which punishment is justified.^83^ Such a change would be both ideological and normative, with major political and policy implications.

I have suggested that the retributive ‘justice’ paradigm, or variations thereof, has failed to engage with value pluralism and has responded inadequately to significant social policy questions regarding the impact of poverty and social inequality on crime and punishment.^84^ This lacuna represents a moral failure of penal policy, with significant implications for social justice in sentencing. It is axiomatic that the state should engage with both diverse and shared manifestations of ‘justice consciousness’, since a meaningful policy engagement with value pluralism requires a conception of ‘justice’ that is capable of responding to the subjectivity of competing moralities.

A disjuncture between penal and social values has been identified in the ethical practice of sentencing within the criminal trial. Johnston argues that the current disclosure and case management regime, with its emphasis on speed and efficiency, has precipitated a fundamental change in the nature of the criminal trial.^85^ As such, the traditional adversarial trial contest, aimed at establishing what may be accepted as ‘facts’ through proof, has gradually been replaced by a more interventionist process focused on ‘managing’ how the ‘truth’ of what took place becomes established.^86^ Thus, early guilty pleas, forced disclosure and judicial intervention have collectively weakened the due process protections of the adversarial trial. As Johnston suggests,^87^ the accused has been unwittingly co-opted into a process in which the burden of proof placed upon the prosecution has been replaced by an administratively driven search for ‘truth’.

These changes to long-established criminal trial practice coincide with a period where the legitimacy of retributive justice has been increasingly questioned,^88^ and where the politics of austerity has spawned policies that have challenged conventional legal and procedural practice. Furthermore, against this backgound, demands for greater empathy and moral engagement in penal practice, symbolised by restorative forms of intervention, have struggled to penetrate the ideological and normative constraints of retributive justice. Hence, the conventional boundaries between substantive and procedural justice have become increasingly blurred. It is argued that these factors have gradually destabilised existing ethical practice, allowing conflicting system interests and priorities to undermine its legitimacy.

Moreover, increased emphasis on process and outcome has facilitated the introduction of more prescriptive sentencing guidelines, so reducing the judicial discretionary capacity to individualise sentences. Together with factors mentioned earlier, this trend has precipitated changes in ethical practice in what appears to signal the gradual demise of the adversarial mode of trial.^89^ Conflicting professional goals and ethics signify subjective realities, diverse meanings and relative notions of ‘justice’. In practice, these concerns are evidenced in the changing roles of the prosecution and the defence,^90^ and the increased impact of formal and informal plea agreements on discretionary sentencing decisions.^91^ This gradual collapse of process, procedure and long-standing evidential rules due to austerity and the prioritisation of efficiency goals over substance is likely to strengthen prosecutorial control and, by implication, state control of the criminal process.

A ‘truth’ extracted and processed to satisfy values of resource management and bureaucratic efficiency symbolises a further distancing of the trial paradigm from the kind of structure capable of delivering socially valued solutions to crime problems. Fundamentally, any ‘legitimate’ authority for depriving citizens of their liberty diminishes where the values that give meaning and relevance to state penality begin to lose common attachment. Ultimately, it becomes vital to re-establish that common purpose in order to prevent the breakdown of ‘civil society’. It is argued that this kind of social breakdown could be reversed by revalidating penal ideology to reflect values that are more likely to promote social cohesion.

Sentencing decisions are unlikely to be perceived as fair or ‘just’ merely by strengthening respect for procedural norms. On the contrary, the notion of ‘substantive justice’ suggests a more profound engagement with penal values and ethical practice, one that engages with shared moral objectives.^92^ Accordingly, the structures and norms of sentencing should be informed by values which promote a more flexible normative framework, one that is sufficiently receptive to interventions and outcomes that are meaningful for all those who demand ‘justice’.

### C. Social Justice and the Search for ‘Truth’

In essence, citizens’ perceptions subjectify the value attached to state-sponsored ‘justice’. Accordingly, such perceptions define the kind of ‘truth’ being sought in order to satisfy particular claims. However, what constitutes ‘truth’ and the nature of its construction through trial and sentencing is complex. ‘Truth’ is closely connected to the sense of ‘justice’ and so the ‘legitimacy’ of what takes place; its complexity is reflected in the fact that it may be evaluated in many different ways. This reflects the inherent subjectivity of ‘justice’ and ‘legitimacy’ as constructs and the difficulty of drawing objective conclusions about their collective dimensions.^93^

For individuals, the ‘truth’ of the trial may depend upon its perceived procedural fairness, as much as the substantive outcome; in fact, the two may merge. Normatively, the perception of ‘truth’ is closely linked to that of closure. Conceiving of penalties predominantly in terms of closure suggests a sentencing practice capable of realising values that foster emotional repair and healing. However, it is important to recognise that notions of emotional repair and healing have collective as well as individual dimensions. As Cotterrell argues, the role of the emotions in strengthening bonds of community is likely to be considerable.^94^ ‘Bonds of community’ of varying degrees of stability or fluidity, transience or permanence arise from common or convergent interests, shared beliefs or ultimate values, co-existence in particular cultural or physical environments,^95^ or emotional allegiances. Regrettably, the diversity and polarisation of moral values characteristic of late modern societies, coupled with increased secularisation, may well have increased suspicion of, and alienated, groups and communities (religious or otherwise) with divergent moralities. Hence, whilst understandings of ‘truth’ may diverge, its meaning remains firmly rooted in the social reality of citizens’ lives. Objectively, the search for ‘truth’ and social justice is a crucial moral *and* political question with no settled answer.

If conventional forms of punishment do not satisfy demands for ‘truth’, it is unclear which alternative approaches the state should pursue. One might consider the possible ambit of du Bois-Pedain’s suggested ‘reasonable terms’ for state intervention,^96^ and what this could mean in practice. One possibility is that it represents a ‘truth’ which satisfies the polity’s idea of ‘justice’, rather than one which engages in moral terms with the values, interests and sensibilities of offenders, victims and communities. Such a conclusion would suggest the need for some tangible and meaningful connection between the abstract philosophical values and justifications for punishment the state might adopt and their physical manifestation in normative and ethical practice. So, whilst the adoption of human rights values might suggest particular moral foundations for penality, sentencing practice must be capable of translating these moral ideals into a meaningful social reality, one that is valued by citizens. I have argued that judicial discretionary power could become a potent tool in helping to achieve this objective.^97^

Whilst Tata emphasises the performative and communicative power of sentencing,^98^ he also explains why ‘social structural *commonalities* … tend to be marginalised’.^99^ Tata’s conclusions suggest that a new paradigm is needed to deliver a form of penal accountability which values punishment for the common good.^100^ This approach is consistent with the view that the processes and structural determinants of judicial reasoning and discretionary decision making within the trial are crucial in shaping sentencing outcomes. It implies a conceptualisation that is practically purposeful, one where the values informing policy and structure are negotiated in ways which promote meaningful connections between the institutions of punishment and citizens.^101^

In sum, an appreciation of judicial discretionary power as potentially instrumental and transformative within the trial process is crucial for developing interventions such as restorative justice that resonate with the concerns of victims and communities. Enhanced discretion and procedural flexibility would allow sentencers to engage more effectively with factors that shape perceptions of ‘justice’ within diverse communities. Most importantly, in helping to reconcile the state’s idea of ‘justice’ with that of citizens and communities, such changes could prove instrumental in advancing social justice more generally.

### D. Restorative Solutions and Social Harmony

Du Bois-Pedain suggests that the decision-making context of sentencing is essentially that of moral interaction, in the sense that the sentencer makes a moral decision and takes moral responsibility for the sentence imposed within the constraints imposed by law.^102^ The significance of this process cannot be overstated. In considering ideas of juristic responsibility, Cotterrell re-examines Radbruch’s ideas about the role of the jurist as a moral guardian for law, a guardianship where moral responsibility does not slavishly follow the state’s chosen value orientation, but one that reflects upon the idea of ‘justice’ and law’s purpose; ideas that resonate with those of Dworkin’s conceptualisation of law’s ‘integrity’.^103^

Cotterrell interprets Radbruch’s focus on law as moral guardianship as one that prevents any externally imposed value system from dominating law’s normative practice. Thus, working within law’s criteria and evaluative methodology, the jurist’s moral responsibility is to ‘work out an independent practical meaning for the idea of law and the realisation of that idea in particular contexts’. Correspondingly, within existing constraints, one might argue that a crucial aspect of the work of the sentencing judge is to recognise the moral value of punishment in a particular context and to give practical meaning to that value in sentencing.

Cotterrell also acknowledges the importance of the jurist’s role in rationalising conflict and preserving ‘law as a universal good in the face of moral and political disagreement’.^104^ Thus, one may argue further that allocating value to law in a way that best discharges the judge’s moral responsibility to reflect social value is to make decisions that resonate with citizens’ perceptions of ‘justice’. Such decisions may increase legitimacy; they are more likely to strengthen ‘bonds of community’ by promoting social cohesion through effective desistance and reintegration. This kind of approach not only acknowledges the judicial capacity to signify law’s moral virtue in maintaining order, it goes much further by suggesting the need for that morality to be contextualised for the common good, thus grounding the morality of law in the social fabric of civil society.

An important aspect of the argument advanced herein is that judicial discretionary power has significant potential as a means of facilitating restorative solutions to crime. To date, the philosophical and normative straitjacket of retributive penal values has largely frustrated attempts to mainstream restorative interventions. More recently, O’Mahony and Doak have developed notions of agency and empowerment as a way of reimagining and justifying the use of restorative justice.^105^ Empowerment through increased agency is viewed as key to enhancing the autonomy and dignity of the individual. Accordingly, the value of individual autonomy changes where individuals are able to accept responsibility for their actions and seek to put things right. This kind of accountability suggests that individuals are only able to create and accept obligations and commitments which recognise the impact and consequences of crime on the self *and* others. Individuals should therefore be encouraged to make informed choices and play an active role in decision making because it may have positive impacts for both the individual and others. Agency shapes goals and outcomes, and so accountability in terms of obligations and commitments. Such an approach fosters social values of inclusion, security and solidarity, values that encourage individuals to accept responsibility for the consequences of their crimes whilst acknowledging its social embeddedness.

The sense of common justification is enhanced through empowerment and agency of both the victim and the accused. Crucially, empowerment and agency must exist within the framework of a value system that is shared, supported and valued by the community to be effective. The moral justification for restorative justice resolution is key to realising this imperative; it reaches beyond the immediate parties to the community itself, so that the empowerment and agency of the parties engages directly with the community in a process of social as well as personal healing. If, as argued, that process is encapsulated within a transformed trial, made possible through a changed penal ideology grounded in shared values, the ‘truth’ sought through process and procedure will have a common focus and direction based upon shared purposes and values. Thus, the search for ‘truth’ will possess the shared ‘meaning’ and ‘relevance’ it currently lacks.

As argued, what is proposed would establish both a communitarian and a political basis for asserting the ‘legitimate’ authority of state penality. The communitarian basis for this authority derives from the identification and adoption of a shared value system to underpin state penal ideology. Correspondingly, the political legitimacy of state penality and its dependent structures stems from more direct engagement with citizens and communities to ascertain the social value of penal interventions and outcomes. In this sense, sentencing could become the driver for a more socially democratic form of penal empowerment, one that reflects contextualised notions of individual and social accountability for offenders, victims and victim communities.

In abstract terms, restorative justice may be conceived as a vehicle for realising the terms of a particular penal relationship between citizen and state, the authority for which derives from the polity. Ethically, the most significant advantage of restorative practice is its emphasis on individual and community empowerment, essentially because this gives the parties a central role in shaping both process and outcome. Accordingly, restorative intervention may be conceived as a process which strengthens social cohesion. Restorative intervention empathises with the restorative values underpinning the process and its aims; it signifies a sympathetic and motivational engagement where participants are empowered to realise restorative values. Thus, restorative agency becomes the vehicle for the democratic empowerment of individuals *and* communities, facilitating ‘justice’ within a secure and supportive space where restorative values are more likely to be realised for the common good.

## 8. Sentencing as a Communitarian Practice

I have argued that the values underpinning sentencing policy in England and Wales need to change fundamentally to enhance social justice in sentencing. This aspiration is a significant departure from deserts-based ‘justice’, which prioritises censure, proportionality, and consistency over social aims. In abstract terms, the notion of ‘social justice’ suggests a commitment to equality, inclusivity, and non-discrimination, and, by implication, a sentencing system which values penal intervention as a social good.

The Lammy Review again highlighted the urgent need to re-establish trust in criminal justice.^106^ This lack of trust suggests that the moral credibility of the entire system needs to be restored. Such a fundamental change demands values that can build trust by bringing people together. Sentencing has a crucial role to play in achieving this objective. Instead of being perceived as a route into one narrowly conceived ‘justice’ paradigm, sentencing could become a major focus for developing restorative interventions and promoting community-based alternatives to imprisonment. However, trust can only be established if citizens empathise with what the state is trying to achieve.^107^ This kind of empathy is essentially moral—the perception that what the state does in the name of ‘justice’ is valued for reasons of the common good.^108^

The British Academy Report drew attention to the need for penal policy to promote the following ‘core’ values for each citizen: namely, liberty, autonomy, solidarity, dignity, inclusion and security, values that ‘play a central role in the way in which contemporary democracies understand themselves (as evidenced by the ways in which aspects of them figure in international human rights conventions)’.^109^ Hence, these values would, if adopted, inform attempts to formulate new options for punishment, reserving imprisonment for only the most serious cases.^110^ However, the Report did not go far enough in suggesting how to bring about a shift towards a more socially ‘just’ kind of penal policy.

Such a fundamental change in the values that underpin sentencing policy and practice could be achieved without destabilising the coherence of the system. Reconceiving the moral foundations for sentencing means that existing justifications would take on new meanings and fulfil different aims. Thus, retributive values would be regarded as *part* of a range of possible justifications for penal intervention, not the primary motivator. Taking greater account of the relationship between social factors and criminality whilst recognising the wider social impact of sentencing decisions on desistance and reintegration should bolster the moral credibility of sentencing, increase social value at the community level and promote social cohesion. Broadening the justificatory basis for penal intervention by adopting core values that promote social justice should encourage parsimony in the use of imprisonment and facilitate a criminal process that is far more engaged with the social realities of crime and victimisation. Crucially, such core values would be shared by citizens in a ‘real’ sense, so that sentencing decisions empathise with the moral sensibilities of different cultures and communities. The closeness of this connection is fundamental to establishing trust and confidence in the sentencing process and investing it with a greater degree of social legitimacy.

Significant changes to structure and practice would be required to fully integrate restorative interventions into the mainstream sentencing system.^111^ Following value change, a transformed role for sentencing within the criminal trial is proposed. This would provide: a more flexible process attaching equal priority to mediated and restorative responses to crime; changed evidential rules resulting in a factual basis for sentencing that takes more account of social factors; and new objectives and principles to ensure that social impact evidence relevant to the sentence is given greater consideration during the trial phase.^112^ Further reforms would include: increased opportunities for judicial intervention and diversion from the established process through enhanced discretionary power; modified rules for widening victim and community access and representation subject to judicial discretion; and merging the sentencing and enforcement phases of the criminal process so that the social impact of penal intervention is recognised and exploited at sentencing and beyond.

To sum up, the proposed reforms would reposition judicial discretion within the framework of a reconceived penal ideology based on shared values. Whilst fundamental, I would argue that the proposed changes are neither impossible to achieve nor politically unrealistic. As currently conceived, sentencing decisions can damage public confidence and weaken trust in the criminal justice system.^113^ This, in turn, weakens the effectiveness of penal measures and threatens social cohesion. The perception of decisions as discriminatory diminishes their social value and the perceived legitimacy of state penality. Fundamentally, this socially destructive spiral reflects a moral failure of ideology and policy. However, it is primarily a political failure. A coherent link between sentencing and social justice is absent because neither ideology nor policy currently addresses the problem; such considerations are considered beyond the remit of sentencing as presently conceived—they are essentially political choices to be argued for.^114^

Until now, sentencing policy and social policy have endured an uneasy relationship and remain largely distinct. Proponents of this view argue that sentencing should focus on delivering a principled and consistent response to behaviour that has been criminalised by the state. Accordingly, allowing judges and magistrates greater discretion in deciding what weight to allow social factors in mitigation is a recipe for inconsistency and incoherent sentencing. Easton argues that compensatory justice at the point of sentencing is a ‘blunt instrument’, incapable of resolving what are deep-seated social injustices and inequality.^115^ So, the best way forward for sentencing is to endeavour to be as even-handed as possible, to avoid discrimination by adopting a rights-based approach, rather than widening the parameters for exercising judicial discretion.

This is an outdated and narrow vision. A sentencing policy committed to social justice should encourage rather than restrict the socially constructive exercise of judicial discretionary power. This means opening up sentencing to a far greater dialogue with communities about social impact, particularly its wider potential for promoting desistance and reintegration, and strengthening social cohesion. Ultimately, the mobilisation of judicial discretion requires a cooperative project to reposition the ideology of sentencing. Such a redirection of judicial discretionary power will only become possible if appropriate normative structures are developed from the core ideological changes proposed.^116^ Once at liberty to look beyond retributive justice, the judiciary could become the driving force for developing crucial new areas of communitarian penal intervention.^117^ Such a move would fundamentally alter the moral terms upon which the state justifies the penal response to crime and criminality.

## Footnotes

1 This approach is consistent with the liberal idealism of Becarria; see C Beccaria, *Of Crimes and Punishments and Other Writings* (first published 1764, R Bellamy ed, CUP 1995).

2 The primary focus of this article is the sentencing policy and practice of England and Wales, although the arguments put forward are intended to be more generally applicable.

3 Moral individualism asserts that the common humanity linking all human beings has a social value. However, social conditions determine the degree to which this value is shared and the extent to which the law respects the autonomous right of all human beings to life and liberty.

4 For excellent commentary, see R B M Cotterrell, *Emile Durkheim: Law in a Moral Domain* (Edinburgh UP 1999).

5 This article adopts Garland’s definition of ‘penality’ as meaning: ‘the networks of laws, processes, discourses, representations and institutions which make up the penal realm’ (D Garland, *Punishment and Modern Society* (Clarendon Press 1990) 17); ‘penality communicates meaning not just about crime and punishment but also about power, authority, legitimacy, normality, morality, personhood, social relations, and a host of other tangential matters’ (ibid 252).

6 A crucial point here is that the values underpinning sentencing policy should be shared for reasons of the common good. See further R Henham, *Sentencing Policy and Social Justice* (OUP 2018) ch 4. For seminal earlier contributions, see B Hudson, *Penal Policy and Social Justice* (Macmillan 1993); B Hudson, ‘Doing Justice to Difference’ in A Ashworth and M Wasik (eds), *Fundamentals of Sentencing Theory* (Clarendon Press 1998). Common good values are those which prioritise respect for human dignity as essential for maintaining social harmony and working towards social improvement for the benefit of all citizens. An important aspect of this conception is the notion that respect for human dignity and the life and security of the individual are values which intersect one another. However, ‘common good’ values are not necessarily the same thing as ‘shared’ values. Values may be shared for reasons that do not benefit the good of all; to further hegemonic ambitions, for example. Common good values are values that benefit *all* members of society.

7 ‘Social values’ are the ‘core’ values by which the state justifies punishment through sentencing. However, the way in which such values are *perceived* by citizens and communities is key—in other words, policymakers need to understand what the foundational values of penal ideology signify in reality. Henham (n 6) ch 1.

8 This approach is most commonly associated with the notion of ‘individualism’ in sentencing. See D Thomas, *Principles of Sentencing* (Heinemann 1979). The Criminal Justice Act 1991 replaced the distinction between tariff and individualised sentences with a statutory deserts-based framework for sentencing which significantly curtailed the judicial capacity to individualise sentences.

9 C Tata, *Sentencing: A Social Process, Re-thinking Research and Policy* (Palgrave Macmillan 2020) ch 2.

10 See A Bottoms and J Tankebe, ‘Beyond Procedural Justice: A Dialogic Approach to Legitimacy in Criminal Justice’ (2012) 102 J Crim L & Criminology 119.

11 See Henham (n 6) ch 4.

12 This assumes that social harmony is valued more than social conflict.

13 See Henham (n 6) 122–39.

14 For fuller discussion of Durkheim’s thinking on the relationship between law and morality, see Cotterrell, *Emile Durkheim* (n 5) 53.

15 R B M Cotterrell, ‘Introduction: Durkheim on Justice, Morals and Politics’ in R Cotterrell (ed), *Emile Durkheim: Justice, Morality and Politics* (Routledge 2010) xi–xxiv <https://papers.ssrn.com/sol3/papers.cfm?abstract_id=1620127>.

16 For further discussion, see British Academy Report, *A Presumption Against Imprisonment: Social Order and Social Values* (The British Academy 2014) 70.

17 ‘Social value’ refers to the value that citizens and communities *actually* place on specific penal interventions like sentencing—the issue here is the degree to which the state’s values are translated into something that has ‘real’ social worth. In short, the difference between what the state desires and thinks it is achieving and what is achieved in practice.

18 Cotterrell, ‘Introduction’ (n 15).

19 R Cotterrell, ‘Theory and Values in Socio-Legal Studies’ (2017) 44 (S1) Journal of Law and Society S19–S36.

20 Similarly, a shared belief in the moral virtue of criminalisation should exist.

21 ‘Secularisation’ is used here in a non-religious sense to indicate the drifting apart or fracturing of social morality.

22 Arguably, this should consist of *all* citizens within ‘civil society’.

23 See eg R B M Cotterrell, ‘Justice, Dignity, Torture, Headscarves: Can Durkheim’s Sociology Clarify Legal Values?’ (2011) 20 Social & Legal Studies 3.

24 For example, the widespread use of capital punishment in England during the era of the ‘Bloody Code’. See V A C Gatrell, *The Hanging Tree: Execution and the English People 1770–1868* (OUP 1994).

25 See Cotterrell, ‘Introduction’ (n 15).

26 ibid 15.

27 ibid 17.

28 ibid 17.

29 See J Weinrib, ‘Dignity and Autonomy’, *Max Planck Encyclopedia of Comparative Constitutional Law* (2019) <https://ssrn.com/abstract=3336984>.

30 On the relationship between beliefs and punitive reactions towards offending behaviour, see K Fousiani and J-W van Prooijen, ‘Reactions to Offenders: Psychological Differences between Beliefs versus Punishment’ (2019) 58 British Journal of Social Psychology 894.

31 See Henham (n 6) ch 4.

32 The meaning and relationship of value concepts in the ethical practice of criminal justice remain relative to time and place. See G Watson, *Respect and Criminal Justice* (OUP 2020) ch 1.

33 I Koch, *Personalizing the State: An Anthropology of Law, Politics, and Welfare in Austerity Britain* (OUP 2018).

34 I Loader, ‘Fall of the Platonic Guardians: Liberalism, Criminology and Political Responses to Crime in England and Wales’ (2006) 46 Brit J Criminol 561.

35 Community sentences are a case in point since they may be justified in many different ways.

36 See Watson (n 32).

37 See R Williams, *Faith in the Public Square* (Bloomsbury 2012) 2, 3 and his discussion of ‘programmatic secularism’.

38 See Cotterrell, ‘Introduction’ (n 15).

39 N Hutton, ‘Sentencing, Inequality and Justice’ (1999) 8 Social & Legal Studies 577.

40 S Easton, ‘Dangerous Waters: Taking Account of Impact in Sentencing’ [2008] Crim L R 105.

41 See The Lammy Review, *An Independent Review into the Treatment of, and Outcomes for, Black, Asian and Minority Ethnic Individuals in the Criminal Justice System* (2017) <https://assets.publishing.service.gov.uk/government/uploads/system/uploads/attachment_data/file/643001/lammy-review-final-report.pdf>.

42 See Henham (n 6) ch 7.

43 Gelsthorpe and Padfield define ‘discretion’ as ‘the mandated or assumed ability of an actor to choose among alternative courses of action or inaction’. See L Gelsthorpe and N Padfield, ‘Introduction’ in L Gelsthorpe and N Padfield (eds), *Exercising Discretion: Decision-Making in the Criminal Justice System and Beyond* (Willan Publishing 2003); R Pattenden, *The Judge, Discretion and the Criminal Trial* (OUP 1982) ch 1; K Hawkins, *The Uses of Discretion* (Clarendon Press 1995).

44 See Tata (n 9) ch 2.

45 See S R Anleu, R Brewer and K Mack, ‘Locating the Judge within Sentencing Research’ (2017) 6 International Journal for Crime, Justice and Social Democracy 46, 55.

46 However, quantitative scholars such as Jose Pina-Sanchez have made a significant contribution in operationalising sentencing concepts <https://scholar.google.co.uk/citations?user=4nfr4tQAAAAJ&hl=en>.

47 Rules of procedure and practice directions are similarly fixed.

48 Moreover, individual judges and magistrates must establish a clear understanding of the relevant law before applying it to the instant case.

49 J Hogarth, *Sentencing as a Human Process* (University of Toronto Press 1971).

50 R Cotterrell, *Sociological Jurisprudence: Juristic Thought and Social Inquiry* (Routledge 2018) 20–4.

51 Henham (n 6) 116. Note that Raz does not support any connection between the morality of punishment norms and the morality of their consequential application by the institutions of punishment. J Raz, *Ethics in the Public Domain* (OUP 2001) 333.

52 RA Duff, *Punishment, Communication and Community* (OUP 2001).

53 D Garland, *The Culture of Control: Crime and Social Order in Contemporary Society* (OUP 2001).

54 The purposes of sentencing as originally set out in the Criminal Justice Act 2003, s 142 (now the Sentencing Act 2020, s 57(2)) contain no overriding rationale or means for prioritising or reconciling the different purposes for sentencing. See A Ashworth, ‘Criminal Justice Act 2003 (2) Criminal Justice Reform: Principles, Human Rights and Public Protection’ [2004] Crim L R 516, 528.

55 As exemplified by the Offences Against The Person Act 1861.

56 Tata (n 9) 57.

57 Gelsthorpe and Padfield make the point that the implementation of rules requires an interpretation of both ‘rules’ and ‘facts’ (n 43).

58 Tata (n 9) 58.

59 ibid 69.

60 ibid 70.

61 Hutton states: ‘Law does not regulate practice it regulates accounts of practice. We can only have access to accounts of practice and to the outcomes of practice, we cannot have access to the internal cognitive processes of individual decision-makers.’ See N Hutton, ‘Visible and Invisible Sentencing’ in A Hondeghem, X Rousseaux and F Schoenaers (eds), *Modernization of the Criminal Justice Chain and the Judicial System: New Insights on Trust, Cooperation and Human Capital* (Springer 2016) 158.

62 Henham (n 6) ch. 4.

63 A du Bois-Pedain, ‘Punishment as an Inclusionary Practice: Sentencing in a Liberal Constitutional State’ in A du Bois-Pedain, M Ulväng and P Asp (eds), *Criminal Law and the Authority of the State* (Hart Publishing 2017).

64 ibid Part III.

65 Henham (n 6).

66 Williams (n 37).

67 A du Bois Pedain, ‘In Defence of Substantial Sentencing Discretion’ (2017) 28 Crim L F 391, 395.

68 ibid 423–7.

69 ibid 433–4.

70 For an alternative perspective, see D Hayes, *Confronting Penal Excess: Retribution and the Politics of Penal Minimalism* (Hart Publishing 2019); see also the review by N Padfield [2020] Crim L R 655.

71 See H Riddle, ‘The House of Commons Justice Committee Follow-Up Report on the Role of the Magistracy: What It Says (and What It Does Not Explain)’ [2020] Crim L R 194.

72 ibid 195.

73 Notions of moral and criminal responsibility for crime rarely coincide.

74 Typically, urban communities are culturally, racially or ethnically diverse, with various kinds of overlapping social networks based upon religion, ethnicity, kinship, linguistic heritage, or shared history or traditions.

75 Tata (n 9) 69.

76 This includes establishing the factual basis for sentencing, a task judges are frequently faced with. See M Wasik and A Ashworth, ‘Issues in Sentencing Procedure’ [2020] Crim L R 397, 410.

77 Tiarks states: ‘The use of algorithms in sentencing is more likely to exacerbate bias, decrease transparency and thus decrease penal legitimacy overall.’ See E Tiarks, ‘The Impact of Algorithms on Legitimacy in Sentencing’ (2021) 1 Journal of Law, Technology and Trust 1, 22.

78 This process had already begun. The pandemic merely exacerbated pre-existing weaknesses and accelerated their destructive consequences.

79 D Ormerod, ‘Coronavirus and Emergency Powers’ [2020] Crim L R 473.

80 See Sentencing Academy, ‘Sentencing and the Pandemic: What Are the Options?’ (2020) 2 Sentencing News 8.

81 An early example is the guilty plea, which was originally conceived as a mitigating factor; see Thomas (n 9). For criticism, see J Peay and E Player, ‘Pleading Guilty: Why Vulnerability Matters’ (2018) 81 MLR 929.

82 D De Girolamo, ‘The Mediation Process: Challenges to Neutrality and the Delivery of Procedural Justice’ (2019) 39 OJLS 834.

83 See eg the problem of measuring sentence severity: E van Ginneken and D Hayes, ‘“Just” Punishment? Offenders Views of the Meaning and Severity of Punishment’ (2017) 17 Criminology and Criminal Justice 62.

84 Henham (n 6) ch 8. See also M Tonry, ‘Can Deserts Be Just in an Unjust World?’ in A P Simester, A du Bois-Pedain and U Neumann (eds), *Liberal Criminal Theory: Essays for Andreas von Hirsch* (Hart Publishing 2014). For a liberal defence of just deserts, see A von Hirsch and A Ashworth *Proportionate Sentencing: Exploring the Principles* (OUP 2005).

85 E Johnston, ‘The Adversarial Defence Lawyer: Myths, Disclosure and Efficency—a Contemporary Analysis of the Role in the Era of the Criminal Procedure Rules’ (2020) 24 International Journal of Evidence and Proof 35.

86 See further J Doak and others, *Mapping the Changing Face of Cross-Examination* (2021) <www.ntu.ac.uk/research/groups-and-centres/projects/mapping-the-changing-face-of-cross-examination>.

87 Johnston (n 85) 55.

88 R Canton, *Why Punish?: An Introduction to the Philosophy of Punishment* (Red Globe Press 2017).

89 J Doak and others, ‘Procedural Traditions in Flux’ (2016) 20 International Journal of Evidence and Proof 263.

90 A Britton, ‘Pressing for Sentence? An Examination of the New Zealand Crown Prosecutor’s Role in Sentencing’ (2018) 29 Crim L F 377.

91 Tata (n 9).

92 See R Canton, ‘Crime, Punishment and the Moral Emotions’ Righteous Minds and their Attitudes towards Punishment’ (2015) 17 Punishment and Society 54.

93 J W de Keijser, L Noyon and J H Crijns, ‘ Legitimacy and Public Opinion: A Five-Step Model’ (2020) 1 International Journal of Law in Context 390.

94 R B M Cotterrell, ‘The Concept of Crime and Transitional Communities: A Perspective from Socio-legal Theory’ (WG Hart Legal Workshop on Globalisation, Criminal Law and Criminal Justice, Institute of Advanced Legal Studies, University of London, 2012) 10.

95 This includes racially distinct or ethnic minority communities, multicultural environments, areas of social deprivation and poverty, and areas of social transition.

96 Du Bois-Pedain, ‘In Defence of Substantial Sentencing Discretion’ (n 67).

97 See further Henham (n 6) ch 7.

98 Tata (n 9) 167–8.

99 ibid 87.

100 Henham (n 6) ch 4.

101 For an excellent illustration of this kind of approach, see Centre for Justice Innovation, ‘Delivering a Smarter Approach: Reforming Out of Court Disposals’ (2020) <https://justiceinnovation.org/sites/default/files/media/document/2020/Delivering%20a%20smarter%20approach.pdf>.

102 Du Bois-Pedain, ‘In Defence of Substantial Sentencing Discretion’ (n 67).

103 Cotterrell, *Sociological Jurisprudence* (n 50) 34–5.

104 ibid 38, 43.

105 D O’Mahony and J Doak, *Reimagining Restorative Justice: Agency and Accountability in the Criminal Process* (Hart Publishing 2017). For more cautionary analyses, see A Ashworth, ‘Responsibilities, Rights and Restorative Justice’ (2002) 42 Brit J Criminol 579; A Ashworth, A von Hirsch and C Shearing, ‘Specifying Aims and Limits for Restorative Justice: A “Making Amends” Model’ in A von Hirsch, J Roberts and A E Bottoms (eds), *Restorative Justice and Criminal Justice* (Hart Publishing 2003).

106 The Lammy Review (n 41).

107 A key element is to increase judicial diversity.

108 Conceptions of the common good generally recognise the importance of social justice. See further Henham (n 6) ch 4.

109 British Academy Report (n 16) 68.

110 The Report notes that the exclusory effects of imprisonment promote social breakdown by destroying the essential fabric of society, the essential ‘bonds of community’ that bind communities together and give them a sense of belonging to a greater whole. ibid 73.

111 See Henham (n 6) ch 7 for detailed explanation.

112 ibid 200–35. The key issues are: which factors should be recognised; what kind of causal link should exist; how should this be demonstrated in court; at what stage in the process; and how to quantify social adversity for the purpose of sentencing. See J Roberts, ‘Contemporary Issues in Sentencing’ (Public Guest Seminar, Institute of Criminology, Cambridge, 2021).

113 Consider the sentencing decisions taken during the 2011 English Riots; see Henham (n 6) ch 2, 71–84. See also J Pina-Sanchez, C Lightowlers and J Roberts, ‘Exploring the Punitive Surge: Crown Court Sentencing Practices before and after the 2011 English Riots’ (2017) 17 Criminology and Criminal Justice 319.

114 H Annison, ‘Politics and Penal Change: Towards an Interpretive Political Analysis of Penal Policymaking’ (2018) 57 Howard Journal of Crime and Justice 302.

115 S Easton, ‘Dangerous Waters: Taking Account of Impact in Sentencing’ [2008] Crim L R 105.

116 Extensive training would be required to accompany such structural changes.

117 An important contemporary example is the theory and practice of ‘social rehabilitation’. See F Coppola and A Martufi (eds), *Social Rehabilitation and Criminal Justice* (Routledge forthcoming).

## Notes

I am most grateful to Professors Roger Cotterrell and Jonathan Doak for their helpful comments on earlier drafts of this article. Needless to say, any errors that remain are mine.

